# *Aronia Melanocarpa* as a Multifunctional Superberry: From Antioxidant Potential to Therapeutic and Nanotechnological Applications

**DOI:** 10.1007/s13668-026-00736-6

**Published:** 2026-02-07

**Authors:** Yasemin Açar Kuru, Gül Eda Kılınç

**Affiliations:** Department of Nutrition and Dietetics, Ondokuz Mayıs University, Samsun, Turkey

**Keywords:** Aronia melanocarpa, Bioavailability, Health benefits, Antioxidant activity, Polyphenolic compounds, Nanotechnology

## Abstract

**Purpose of the Review:**

This review aims to provide an overview of the nutritional composition, bioactive compounds, and health-promoting properties of *Aronia melanocarpa*, with a particular focus on its antioxidant capacity and potential therapeutic applications in chronic disease prevention and management.

**Recent Findings:**

In vitro, in vivo, and clinical studies indicate that *Aronia melanocarpa* exerts potent antioxidant, anti-inflammatory, antidiabetic, cardioprotective, anti-obesity, and anticancer effects, attributed mainly to its rich anthocyanin and procyanidin content. Human intervention trials have shown improved antioxidant status, lipid and glucose metabolism, vascular function, and inflammatory markers. However, some results remain inconclusive due to differences in cultivar, processing, dosage, and individuals. Supporting evidence from animal and cellular models highlights the regulation of NF-κB and AMPK signaling pathways, modulation of gut microbiota, and protective effects against metabolic and age-related disorders. Furthermore, novel applications in nanotechnology demonstrate that *Aronia melanocarpa* extracts can serve as eco-friendly agents in synthesizing bioactive nanoparticles with antioxidant, antimicrobial, and biomedical potential.

**Summary:**

*Aronia melanocarpa* is a promising functional food with strong potential for nutraceutical and therapeutic applications. While experimental evidence supports its health benefits, human clinical data remain insufficient to establish standardized dosage and long-term use recommendations. Future research should focus on well-designed clinical trials, bioavailability optimization, and fully elucidating the role of *Aronia melanocarpa* in health promotion and disease prevention.

## Introduction

Among fruits, berries, in particular, are rich in natural antioxidants and possess high antioxidant capacity and anthocyanin levels [1].

*Aronia melanocarpa* (Black chokeberry) originated in North America and eastern Canada [2]. Three known species are within this genus: *Aronia melanocarpa* (Michaux) Elliot (Black chokeberry), *Aronia prunifolia* (Marshall) Rehder (Purple chokeberry), and *Aronia arbutifolia* (Linnaeus) Persoon (Red chokeberry) [3]. *Aronia melanocarpa* can be consumed fresh today, but due to its bitter taste, it is generally processed into fruit juice, concentrate, jam, dried fruit, tea, wine, liqueur, and other alcoholic beverages in the food industry [4].

Studies on the effects of *Aronia melanocarpa*, a type of berry, on human health have revealed that its fruits have the highest antioxidant capacity and anthocyanin content compared to other berries [5]. Regular consumption of this berry has been found to protect against cardiovascular diseases, digestive system diseases, and some cancers [6]. *Aronia melanocarpa* is a berry that adapts to various climates and soil conditions and contributes significantly to human health and nutrition with its rich composition [7]. The fruit contains very high concentrations of anthocyanins and flavonoids. It is also rich in antioxidants, phenols, minerals, and vitamins. These chemicals are reported to have the potential to prevent heart disease and cancer [8]. *Aronia melanocarpa* berries have been recognized as medicinal plants due to their biochemical properties. It may have beneficial effects on various diseases, including colds, stomach ailments, intestinal diseases, liver and gallbladder diseases, and radiation poisoning [9]. *Aronia melanocarpa* also has a high antioxidant capacity that nourishes the brain and nervous system and helps combat aging [10].

Although *Aronia melanocarpa* is recognized for its exceptionally high antioxidant capacity and rich anthocyanin content, the clinical translation of these bioactive compounds remains challenging. Anthocyanins are highly susceptible to degradation during digestion and exhibit limited bioavailability, which may attenuate their therapeutic effects related to oxidative stress modulation and inflammation control [5, 7–10]. In this context, nanotechnology-based delivery systems have emerged as promising approaches to enhance the stability, bioaccessibility, and biological efficacy of *Aronia melanocarpa* phenolics, thereby strengthening their therapeutic relevance [11–13].

This review aimed to synthesize current evidence on the digestive stability, health-related effects, and pharmaceutical applications of *Aronia melanocarpa* bioactive compounds, with particular emphasis on the determinants of anthocyanin bioavailability. Additionally, the review critically examined emerging nanotechnology-based approaches developed to enhance anthocyanin stability and optimize their potential bioavailability.

## Composition and Bioactive Contents of *Aronia Melanocarpa*

The chemical components of *Aronia melanocarpa* fruit have been reported to include nutrients (carbohydrates, organic acids, protein, lipids, vitamins, minerals, etc.) and phenolic compounds [14]. Other components, such as phenolic compounds, organic acids, proteins, and lipids, affect *Aronia melanocarpa* fruit quality and stability. A literature review has revealed significant variation in the amount and distribution of these components. These differences have been reported to depend on genetic factors such as species and cultivar, as well as environmental factors such as maturity, harvest time [15], fertilization [16], irrigation, climate, light, temperature, and location [17].

Although the dry matter ratio of *Aronia melanocarpa* berries varies between studies, it has been reported to be between 17 and 31% [18–20]. It has been reported that the pH values ​​of *Aronia melanocarpa* berries vary between 3.3 and 3.7, with malic acid being the dominant acid, though they have a lower concentration of organic acids compared to other berries [20, 21]. Fresh *Aronia melanocarpa* fruit has been reported to have a total fat content that varies between 0.09% and 0.17% in the analyses. It has been reported that a significant amount of the lipids found in *Aronia melanocarpa* fruit are sterols and phospholipids, which make up 13.9% of the lipids found in the seed. The remaining lipids are found in the pulp (2.9–9.8%), where it has been reported that 90.49% of the total fatty acids consist of unsaturated fatty acids and 9.51% of saturated fatty acids. Among these fatty acids, the most abundant are linoleic (C18:2), followed by oleic acid (C18:1), and these fatty acids constitute 43.43% and 16.38% of the fatty acid composition, respectively [4]. The carbohydrates of *Aronia melanocarpa* are sugars and dietary fiber, and the total carbohydrate content of fresh *Aronia melanocarpa* fruit has been reported to vary between 6.21 and 20.9% [17]. As in other berries, the amount of protein and amino acids in *Aronia melanocarpa* berries is relatively low. A study has reported the protein content of *Aronia melanocarpa* berries as 0.60–0.81% based on fresh fruit weight [22]. *Aronia melanocarpa* berries contain essential and nonessential amino acids, including arginine, tyrosine, histidine, lysine, cysteine, alanine, asparagine, serine, glutamic acid, and threonine. The most abundant amino acids are threonine with 0.033–0.39 mg/100 g and serine with 0.023–0.39 mg/100 g, respectively [17]. *Aronia melanocarpa* contains macro (essential) minerals such as calcium, magnesium, phosphorus, and potassium, as well as micro (trace) minerals such as iron, copper, iodine, zinc, and selenium [23].

## Antioxidant Properties of *Aronia Melanocarpa*

*Aronia melanocarpa* has been reported to possess significant antioxidant properties, and one study indicated that its high phenolic content is responsible fot this potency. In a study investigating the antioxidant capacities of different berries using the Oxygen Radical Absorbance Capacity (ORAC) method, the highest antioxidant activity value was determined in *Aronia melanocarpa* berries (160.2 µmol of TE/g). The concentration in the sample was reported to be approximately four times higher than the closest sample, blueberry (38.1 µmol of TE/g), and approximately nine times higher than the lowest sample, cranberry (18.5 µmol of TE/g) [24]. In a similar study comparing the chemical composition and antioxidant activity of some small berries (Sambucus, Aronia, Ribes, Hippophae rhamnoides, Rubus), the highest phenolic compound concentration and antioxidant activity were found in Viking, Aron, and Cleata cultivars of the aronia genus [25].

Additionally, a high correlation was found between DPPH and ABTS antioxidant activity results and total phenolic compounds (*r* = 0.98 and *r* = 0.98) and total anthocyanins (*r* = 0.93 and *r* = 0.90) [26]. Thanks to the polyphenols and antioxidant capacity found in *Aronia melanocarpa*, it has been reported to exhibit positive effects on the gut microbiota [27], immunomodulatory properties [28], protection against DNA damage [29], and anti-inflammatory activity [30]. The antioxidant activity of *Aronia melanocarpa* is reported to have effects far beyond scavenging free radicals in vivo. These activities have been reported to suppress reactive nitrogen and oxygen species, increase the recovery of antioxidant enzymes, inhibit prooxidants, and influence cellular signaling to regulate antioxidant levels within the cell [31–33]. Regular antioxidant consumption has shown significant preventive effects under conditions of oxidative stress, and their protective properties in vivo have been reported [29, 34]. Numerous studies examining the antioxidant effects of *Aronia melanocarpa* juice in vivo have reported that phenolic content is related to antioxidant activity [35–37]. Although *Aronia melanocarpa* exhibits high in vitro antioxidant activity, the bioavailability of its phenolic compounds and anthocyanins can be limited due to digestive transformation and metabolism, which may affect their in vivo efficacy [38, 39].

## Digestion and Bioavailability of *Aronia Melanocarpa*

During the digestion of *Aronia melanocarpa* berries, anthocyanins undergo rapid microbial catabolism, with cyanidin-3-O-galactoside being rapidly metabolized to peonidin-3-O-galactoside. The catabolization products are approximately 10 times more bioavailable than anthocyanins in plasma and urine. Anthocyanins and other polyphenol catabolites are extensively metabolized in plasma and urine, with a tmax ranging from 1.0 to 6.33 h [40].

The health effects of phenolic compounds are related mainly to their bioavailability. This bioavailability is primarily determined by its release in the gastrointestinal tract and subsequent intestinal absorption. A study in healthy volunteers examined the bioavailability of a dietary dose of anthocyanins from *Aronia melanocarpa* juice. This study found that approximately 0.25% of the anthocyanins that were consumed were excreted renally within 24 h, mostly in cyanidin metabolites [41]. Several animal and human studies have reported that procyanidins are not absorbed in the intestine, reaching the colon essentially unchanged, where they are metabolized into phenolic acids by the colonic microbiota [42, 43].

Although *Aronia melanocarpa* is rich in anthocyanins and procyanidins, the bioavailability of these compounds is low. On the other hand, some flavonoids and phenolic acids found in *Aronia melanocarpa* may be absorbed at a higher level. The absorption of phenolic compounds is influenced by various factors, including structural stability, food matrix, the presence of the glycoside moiety, and colonic microbial activity [38, 39]. These parameters can lead to differences in bioavailability.

Therefore, a detailed investigation of how *Aronia melanocarpa* phenolic compounds are metabolized and absorbed throughout the gastrointestinal tract is necessary. Some in vitro studies have combined luminal digestion models mimicking the upper gastrointestinal tract with Caco-2 cell transport models to assess the bioavailability of anthocyanins, procyanidins, flavonols, and phenolic acids [44–47]. However, the rate of phenolic absorption in the small intestine is relatively low (5–10%); a large portion of these compounds reaches the colon unchanged, where their bioavailability increases due to microbial fermentation [48]. Given the limited bioavailability of these compounds, emerging nanotechnology-based delivery systems have been investigated as potential tools to enhance their stability, absorption, and overall bioactivity [12].

## Nanotechnology Approaches of *Aronia Melanocarpa*

Nanotechnology has emerged as an innovative scientific field in recent years. As a rapidly developing field of research, nanotechnology has numerous applications in various industries, including agriculture and medicine. Due to its low toxicity, biocompatibility, and environmental friendliness, the biosynthesis or green synthesis applications of nanoparticles have recently attracted significant attention [12].

Due to the abundance of environmental and renewable resources, the use of plant extracts to create metallic nanoparticles has become a promising alternative to chemical and physical methods. It possesses a strong antioxidant capacity due to its richness in polyphenolic compounds such as phenolic acids, anthocyanidins, and procyanidins. The presence of such natural components not only offers health-promoting effects but also makes *Aronia melanocarpa* an ideal candidate for green chemistry applications [11]. Although studies have highlighted the bioactivity of *Aronia melanocarpa* and suggested promising interactions at the nanometer scale, there is a lack of information in the current scientific literature regarding its stability, efficacy, and mechanisms of action as a biological agent in nanomaterial development [7, 13, 49]. A summary of studies using *Aronia melanocarpa* in nanotechnology research is presented in Table 1.

**Table 1:** Summary of the literature on the application of *Aronia melanocarpa* in nanotechnology-related studies

Type of Nanoparticles	Type of Aronia melanocarpa	Dose of Aronia melanocarpa	Main Outcomes	References
Zinc Oxide Nanoparticles (ZnO NPs)	*Aronia melanocarpa* berry extract	10 g of berry powder	- *Aronia melanocarpa*-mediated AgNPs are stable, biologically active, and suitable for potential biomedical, cosmetic, and environmental applications, demonstrating the importance of plant-based nanotechnology.	[50]
Zinc Oxide Nanoparticles (ZnO NPs)	*Aronia melanocarpa* extract	5.0 g of powdered *Aronia melanocarpa*	- *Aronia melanocarpa* fruit extract-based phytogenically produced ZnO NPs show great potential for degrading pollutants and could be helpful in various biological applications, such as antibacterial, antidiabetic, and antioxidant activities.	[51]
Tin oxide and titanium dioxide nanoparticles (SnO_2_NPs and TiO_2_NPs)	*Cnicus benedictus* and *Aronia melanocarpa* extracts	10 g of powdered extracts	**-** Using these extracts eliminated the need for harmful reducing agents, offering an environmentally friendly approach compared to traditional green synthesis methods.	[52]
Silver nanoparticles (AgNPs)	*Aronia prunifolia* berry extract	10 g of berry powder	**-** *Aronia prunifolia* leaf extract in the green synthesis of silver nanoparticles, thereby accentuating their viability as antimicrobial agents against fungal pathogens	[12]
Silver nanoparticles (AgNPs)	*Aronia melanocarpa* berry extract	10 g of berry powder	-The produced AgNPs showed antibacterial activity against *Candida albicans*,* E. coli*, and *S. aureus.* Furthermore, based on the outcomes of the ferrous ion chelating assay, hydroxyl scavenging activities, and the lipoxygenase inhibition, AgNPs show encouraging antioxidant potential.	[49]

The studies summarized in Table 1 demonstrate the successful use of Aronia melanocarpa extracts in the green synthesis of various metal and metal oxide nanoparticles such as zinc oxide, silver, tin oxide, and titanium dioxide. In most of these studies, the synthesized nanoparticles are reported to exhibit significant antioxidant, antimicrobial, and catalytic activities. In particular, silver and zinc oxide nanoparticles show increased biological activity thanks to surface modification with Aronia-derived polyphenols, suggesting synergistic effects [49–51].

Furthermore, it is thought that the increased antioxidant effect observed in nanoparticles synthesized with Aronia melanocarpa may be related to the protection and functional presentation of polyphenolic compounds on the nanoparticle surface [11–13]. Findings have been reported that this presentation at the nanoscale can support the scavenging of reactive oxygen species (ROS), chelation of metal ions, and suppression of lipid peroxidation [49]. However, the direct effects of these on oxidative stress-related cellular signaling pathways are not yet sufficiently elucidated [11, 13].

However, some limitations are noteworthy in the current literature. The vast majority of studies are limited to in vitro experiments, and there is limited data on the bioavailability, long-term safety, and in vivo efficacy of Aronia melanocarpa-based nanoformulations. Furthermore, differences in extraction methods, nanoparticle types, and dosages make direct comparisons between studies difficult. This highlights the need for standardized and translational studies, particularly those targeting oxidative stress mechanisms. In conclusion, nanotechnology-based approaches have the potential to enhance the stability and functional efficacy of Aronia melanocarpa phenolics. These strategies, by combining Aronia’s natural antioxidant properties with nanocarrier systems, offer a promising but still developing research area for functional food and therapeutic applications targeting oxidative stress-related processes [11–13, 49–52].

Further, by enhancing the stability, absorption, and biological activity of Aronia melanocarpa phenolics, these nanotechnology-based approaches may further support the potential health-promoting effects of the compounds, bridging their bioactive properties with therapeutic applications.

## Therapeutic and Health Effects of *Aronia Melanocarpa*

The number of studies demonstrating the positive therapeutic effects of *Aronia melanocarpa* berries on some chronic diseases is increasing. *Aronia melanocarpa* berries, rich in bioactive compounds, are used in supportive therapy for diabetes, inflammation, cancer, and cardiovascular diseases [53].

### Effects *of Aronia Melanocarpa* on Obesity and Diabetes

Long-term consumption of *Aronia melanocarpa* extract has been reported to benefit fasting blood glucose levels and lipid profiles [54]. *Aronia melanocarpa* berries may have beneficial effects on glucose metabolism and, therefore, may be considered a potential supportive option in diabetes management. Polyphenolic compounds of *Aronia melanocarpa* (cyanidin 3-rutinoside) reduce blood glucose levels by inhibiting α-glucosidase and α-amylase activities. This may play beneficial roles in preventing diabetes by controlling postprandial hyperglycemia [55]. Similar antidiabetic effects have also been observed in in vivo experimental animal models of diabetes [56]. Another in vitro study showed that *Aronia melanocarpa* extract inhibits the activities of pancreatic α-amylase and lipase, key enzymes of the digestive system [57].

*Aronia melanocarpa* extract reduces risk factors associated with insulin resistance by modulating the insulin signaling pathway and multiple other pathways related to adipogenesis and inflammation. *Aronia melanocarpa* anthocyanins can regulate carbohydrate metabolism in diabetic patients and streptozotocin-diabetic rats [58]. Clinical evidence suggests polyphenol-rich foods modulate carbohydrate metabolism by restoring beta-cell integrity and physiology and increasing insulin secretory activity. Therefore, polyphenol-rich *Aronia melanocarpa* berries may serve as a potential natural option for supporting diabetes management [59]. In this context, 200 ml/day of *Aronia melanocarpa* extract for 3 months has been recommended [60]. *Aronia melanocarpa* berries have been shown to have beneficial effects in reducing oxidative stress and complications due to hyperglycemia as a result of their anti-inflammatory and antioxidant components [61]. *Aronia melanocarpa* fruit extract has also been shown to reduce risk factors associated with insulin resistance by modulating multiple pathways related to insulin signaling, adipogenesis, and inflammation [54]. Pancreatic enzymes such as α-amylase, α-glucosidase, and lipase are essential in converting complex molecules into smaller, more digestible forms during digestion. *Aronia melanocarpa* extracts and their active compounds may offer a practical approach to supporting the management of diabetes and obesity, as they may reduce the bioavailability and energy value of nutrients by inhibiting these enzymes [57, 62]. Clinical studies have shown that *Aronia melanocarpa* fruit juice reduces fasting blood glucose in patients with type 2 diabetes [61, 63]. In a mouse model with diabetes, plasma glucose, body weight, and white adipose tissue were reduced when the mice were given *Aronia melanocarpa* fruit juice. Furthermore, inhibition of DPP IV was achieved. This inhibition may play a role in supporting type 2 diabetes management by potentially enhancing glucose-dependent insulin secretion, slowing gastric emptying, and reducing postprandial glucagon levels and food intake [64].

### Anti-inflammatory Properties of *Aronia Melanocarpa*

The anti-inflammatory properties of *Aronia melanocarpa* berries may play a role in preventing the development of chronic health problems such as diabetes, cardiovascular disease, and immune system disorders. Cyclooxygenase (COX) and inducible nitric oxide synthase (iNOS) are key proinflammatory enzymes responsible for synthesizing lipid mediators and nitric oxide, which are associated with the progression of many inflammatory diseases. *Aronia melanocarpa* extract has been reported to have anti-inflammatory effects on endotoxin-induced uveitis in vivo animal models. Anti-ocular inflammatory effects inhibit the production of nitric oxide, prostaglandin, and tumor necrosis factor-α (TNF-α), resulting from suppressed expression of iNOS and COX-2 enzymes [65]. The decrease in iNOS and COX-2 expression, reduction in radical oxygen species release, and induction of cell cycle arrest prove their anti-inflammatory properties [66]. Studies have also determined that the anti-inflammatory activity is mainly due to the plant’s flavonoid and phenolic acid content. The compounds primarily responsible for the activity were quercetin, cyanidin 3-arabinoside, and hydroxycinnamic acid derivatives [67, 68]. Another study showed that *Aronia melanocarpa* concentrate inhibited the release of TNF-α, IL-6, and IL-8 in human peripheral monocytes and the NF-κB pathway in RAW264.7 macrophage cells. Furthermore, *Aronia melanocarpa* berries were reported to synergize with sodium selenite in inhibiting NF-κB activation, cytokine release, and PGE2 synthesis [30].

### Anticarcinogenic Effects of *Aronia Melanocarpa*

The anticancer properties of *Aronia melanocarpa* berries have been associated with their rich polyphenolic content, which may contribute to the inhibition of tumor cell growth, induction of apoptosis, and protection against carcinogenesis. *Aronia melanocarpa* extract, rich in polyphenolic compounds, induces programmed cell death in T-cell-derived lymphoblastic leukemia cells. Its anticarcinogenic activity is primarily attributed to chlorogenic acids, some cyanidin glycosides, and quercetin derivatives [69]. Numerous in vitro and animal studies have demonstrated antiproliferative or protective effects of *Aronia melanocarpa* berries and their extracts, particularly in colon cancer [20]. Protective effects and multiple mechanisms of action have been determined in colon carcinogenesis [70, 71]. The chemical structure of anthocyanins has been reported to play an essential role in the cytostatic effect [72]. The Ames test determined that anthocyanins isolated from *Aronia melanocarpa* berries possess antimutagenic activity. This activity is thought to occur mainly through free radical scavenging and inhibiting enzymes that activate promutagens and convert mutagens to DNA-reactive derivatives [73].

### Cardiovascular Effects of *Aronia Melanocarpa*

**Fig. 1:**
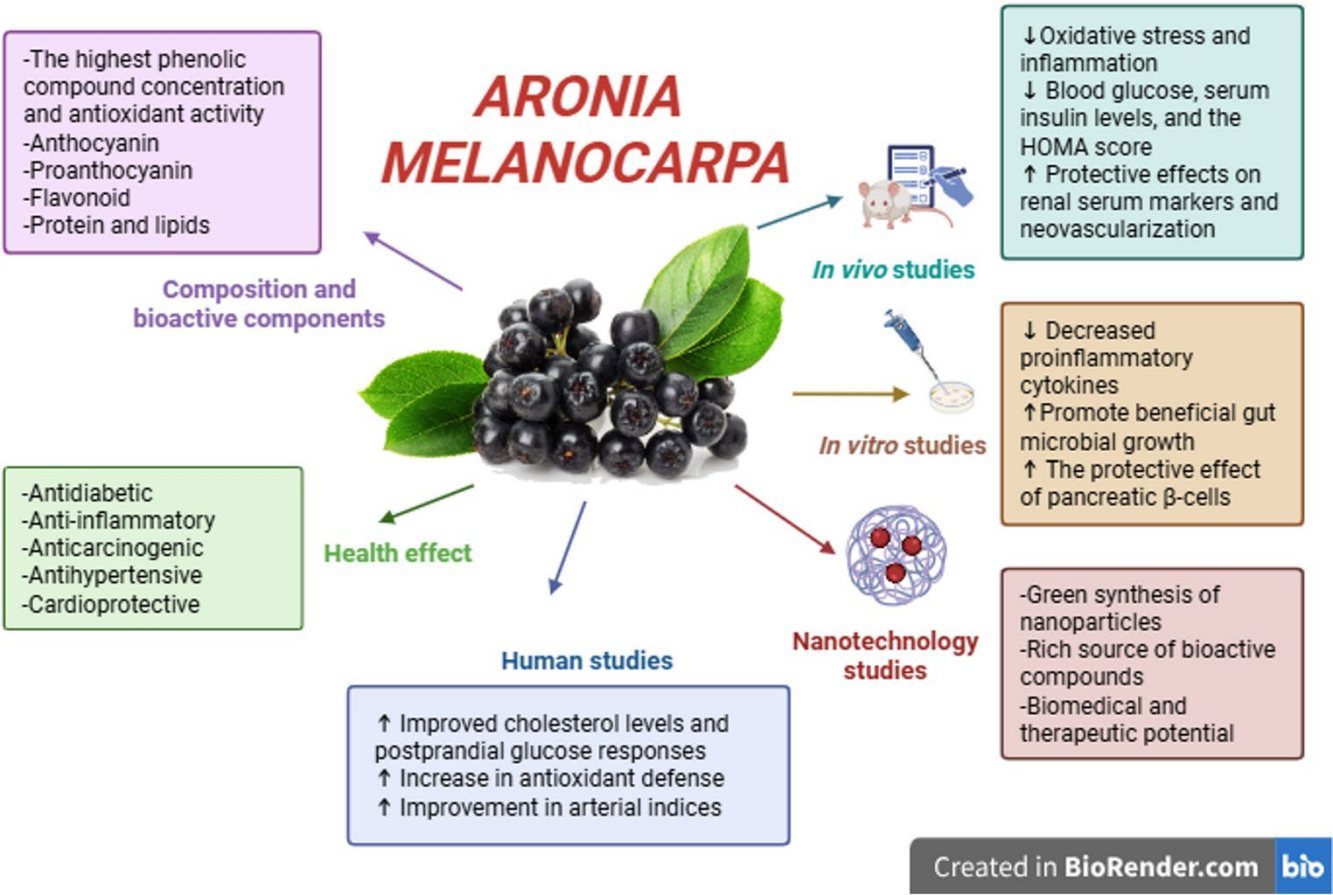
Key physicochemical and functional properties of *Aronia melanocarpa*

Hypertension is a factor that plays a negative role in the development of cardiovascular disease, which is associated with endothelial dysfunction and oxidative stress [74]. Polyphenols can regulate overall cardiovascular health and help manage hypertension due to their ability to reduce vascular oxidative stress. *Aronia melanocarpa* berries exhibit cardioprotective effects due to multiple activities on lipid metabolism, peroxidation, the inflammatory process, coagulation, and oxidation [75]. *Aronia melanocarpa* extract has been reported to reduce induced hypertension in in vivo experimental animal models [76]. This positive effect is based on reducing lipid peroxidation through high total antioxidant capacity. In vivo and in vitro studies report that phenolic compounds protect endothelial cells, contribute to their restoration, and exhibit antiplatelet function [77]. In a study conducted in patients with metabolic syndrome, the use of *Aronia melanocarpa* extract resulted in significant reductions in total cholesterol (TC), low-density lipoprotein cholesterol (LDL-C), and triglyceride (TG) levels. After 1 month of use, a significant inhibition of platelet aggregation was observed, and a reduction in clot formation and fibrinolysis [78]. *Aronia melanocarpa* extract was found to affect the expression of genes involved in cholesterol efflux in Caco-2 cells in a dose-dependent manner, thereby promoting the efflux of cellular cholesterol into the intestinal lumen. This study suggests that the hypolipidemic effects of the extract may be at least partially attributable to increased apical clearance of LDL-C-derived particles and decreased chylomicron formation in the intestine, and that specific isoforms of sirtuin may play an essential role in this process [79]. In another study, rich extracts of *Aronia melanocarpa* fruit caused a significant concentration-dependent reduction in superoxide production only in patients with cardiovascular risk factors. At the same time, no effect was observed in the control group. *Aronia melanocarpa* fruit extracts exhibited a significant concentration-dependent antiaggregatory effect in both study groups, indicating that these effects may be independent of the regulatory effect on superoxide production [80]. Anthocyanin-enriched extracts have been shown to induce endothelium-dependent relaxation in porcine coronary arteries. This suggests that polyphenol-rich extracts, such as *Aronia melanocarpa* extract, may have significant beneficial effects in vascular diseases [81]. The main physicochemical and functional properties of *Aronia melanocarpa* are summarized in Fig. 1.

*Aronia melanocarpa* has recently received significant interest due to its rich bioactive profile and potential health benefits. To better understand its biological effects, in vivo studies, in vitro studies, and animal studies have been conducted. The findings from these investigations are summarized in Tables 2 and 3, and 4, providing a comprehensive overview of *Aronia melanocarpa*’s activities in experimental models and human studies.

**Table 2:** Summary of the protective effects of different *Aronia melanocarpa* types in human intervention studies

Effect on	Characteristics of the Group	Number of Participants (*n*) (Female/Male)	Type of Aronia melanocarpa	Dose of Aronia melanocarpa	Time of Intervention	Main Outcomes	References
Level of antioxidant elements in the serum	Healthy adults	*n* = 102 (67/35)	*Aronia melanocarpa* juice and chokeberry fiber	100 mL and 10 g	90 days	↑ Increase in the levels of copper, manganese, and selenium with antioxidant properties	[82]
Cholesterol, glucose, and serum and gut metabolites	Healthy adults	*n* = 14 (8/6)	*Aronia melanocarpa* juice	100 mL	30 days	↑ Improved total cholesterol levels and postprandial glucose responses ↓ Decrease in inflammatory metabolites	[83]
Gut microbiome and metabolome changes	Healthy, normal-weight females	*n* = 40	*Aronia melanocarpa* juice	100 mL	12 weeks	↑ Increase in lipoprotein profile due to modification of the gut microbiome	[84]
Cardiovascular risk factors	Patients with T2DM	*n* = 59 (23/36)	Fermented or non-fermented *Aronia melanocarpa* extract	34 g	8 weeks	Did not affect waist circumference, insulin sensitivity, or fasting glucose, insulin, or glucagon levels in subjects with T2DM.	[85]
Acute aerobic exercise-induced oxidative stress	Healthy adults	*n* = 70 (35/35)	*Aronia melanocarpa* extract (capsule)	300 mg	8 weeks	↑ Increase in GSH availability and GPx activity	[86]
Inflammatory status and selected markers of iron metabolism	Male football players	*n* = 22	Lyophilized *Aronia melanocarpa* extract	6 g	90 days	Improves the performance and serum antioxidant markers ↑ Increase in the serum TAC and IL-10 levels ↓ Decrease in IL-6 levels	[87]
Cytoprotective and cardiometabolic markers and semen quality	Mildly hypercholesterolemic men	*n* = 109	*Aronia melanocarpa* extract (capsule)	150 mg	90 days	↑ Improved glutathione levels ↑ Increase in the percentage of motile sperm ↓Decrease in sperm DNA fragmentation ↓Decrease in TC and LDL levels	[88]
Arterial function and gut microbiome	Prehypertensive participants	*n* = 102 (55/47)	Chokeberry extract (capsule)	500 mg	12 weeks	↑ Improvement in arterial indices No changes in BP, endothelial function, and blood lipids ↑ Increase in gut microbiome gene richness and in the abundance of butyrate-producing species	[89]
Glycemia, lipid profile, and BP	Patients with MetS	*n* = 144 (74/70)	*Aronia melanocarpa* extract solution	30 mL	4 weeks	↓ Decrease in LDL and TG levels ↑ Positive effect on TC, BP and glycemia	[90]
Redox status	Anemic hemodialysis patients	*n* = 30 (11/19)	*Aronia melanocarpa* extract	30 mL	30 days	↑ Beneficial effects on anemia parameters ↓ Reduce the production of prooxidative molecules ↑ Increase in antioxidant defense	[91]
Cognitive performance, mood, and vascular function	Healthy adults	*n* = 101 (65/36)	*Aronia melanocarpa* extract	90 and 150 mg	24 weeks	↓ Decrease brachial diastolic BP ↑ Improved psychomotor speed Did not affect cognitive flexibility, BDNF, and vascular parameters	[92]
Cardiovascular Risk	Patients at cardiovascular risk	*n* = 84 (52/32)	*Aronia melanocarpa* juice with low- or high-dose of polyphenols	100 mL	4 weeks	Did not affect anthropometric and cardiovascular biomarkers in individuals at cardiovascular risk Altered the composition of plasma phospholipids and fatty acids in ındividuals at cardiovascular risk	[93]
Cardiovascular disease risk, inflammation, and oxidative stress	Healthy adult former smokers	*n* = 49	*Aronia melanocarpa* extract	500 mg	12 weeks	↓ Decrease in fasting plasma TC and LDL levels Did not improve biomarkers of oxidative stress and chronic inflammation	[94]

*BP* Blood pressure, *GPx* Glutathione peroxidase, *GSH* Glutathione, *IL* Interleukin, *TAC* Total Antioxidative Capacity, *BDNF* Brain-derived neurotrophic factor, *MetS* Metabolic Syndrome, *LDL,* *TG,* *TC* Total cholesterol, *T2DM,* Type 2 diabetes mellitus

**Table 3:** Summary of the effects of different *Aronia melanocarpa* types *in vivo/in vitro* models

In vivo/In vitro Models	Characteristics of the Group	Type of Aronia melanocarpa	Dose of Aronia melanocarpa	Time of Intervention	Main Outcomes	References
In vitro	Patients with a diagnosis of hypertension or hypercholesterolemia (*n* = 17)	*Aronia melanocarpa* extract	200 mg/capsule	6 weeks	In the human trial; ↓Significant reductions in total cholesterol, LDL cholesterol, and ALT levels In *in vitro studies;* ↓ Decreased proinflammatory cytokines and adhesion molecules in endothelial cells and peripheral blood cells	[95]
*In vivo and in vitro*	Smoking volunteers (*n* = 9)	*Aronia melanocarpa* fruit juice Water soluble β-escin	5 mg 10 mg	7 days	↓Decrease inflammation associated with tobacco smoking ↑Increase ALDH activity and antioxidant capacity	[96]
In vitro (3T3-L1 preadipocytes) and in vivo C57BL/6 male mice models	Mice fed a high-fat diet	*Aronia melanocarpa* extract	25 and 100 mg/kg	12 weeks	↑Improve blood lipid levels and adipocyte degeneration ↑Promote beneficial gut microbial growth ↑ Activate the AMPK signaling pathway	[97]
In vivo	Male C57BL/6J mice fed a low-fat diet, an HF, and an HS diet, or an HF/HS diet supplemented with *Aronia melanocarpa*	*Aronia melanocarpa* extract	NR	14 weeks	Suppress LPS-induced mRNA expression of inflammation and glycolysis mediators ↑Attenuate obesity-induced AT inflammation by inhibiting NF-κB signaling and the glycolytic pathway in macrophages	[98]
In vitro (T cell cultures) and in vivo C57BL6/J male mice models	Mice fed a control or 4.5% *Aronia melanocarpa* -supplemented diet	*Aronia melanocarpa* extract	6.3 g	4 weeks	↑ Increase colonic IL-10 secretion Do not inhibit ex vivo cytokine secretion of lipopolysaccharide-stimulated spleen and colon tissue. Inhibit TNF-α production in T cells	[99]
In vitro and in vivo C57BL/6 male mice models (*n* = 21)	Mice with chemically-induced diabetes	*Aronia melanocarpa* extract	50 and 200 mg/kg	7 days	↑ Increase in immunomodulatory effects in the gut	[100]
In vitro and in vivo mouse models (male ICR mice) (*n* = 32)	Multiple low-dose streptozotocin-induced T1DM	*Aronia melanocarpa* extract	10 and 100 mg/kg	7 days	↓ Decrease the increase in blood glucose level ↑ The protective effect of pancreatic β-cells	[101]
In vitro	Caco-2 cells	*Aronia melanocarpa* juice	NR	7 weeks	↑ Improve cellular transport of many phenolics in the presence of a colon matrix	[102]

*T1DM* Type 1 Diabetes Mellitus, *ICR,* Institute of Cancer Research, *AMPK* AMP-activated protein kinase, *TNF-a* Tumor Necrosis Factor-a, *NR* Not Reported, *HF* High-fat, *HS* High-sucrose, *LPS,* *AT* Adipose Tissue, *NF-κB* Nuclear factor kappa B, *ALT* Alanine aminotransferase, *ALDH* Aldehyde dehydrogenase

**Table 4:** Summary of the effects of different *Aronia melanocarpa* types in animal models

Animal Models	Type of Aronia melanocarpa	Dose of Aronia melanocarpa	Time of Intervention	Main Outcomes	References
Male Sprague-Dawley rats fed a high-fat diet	Microencapsulated extract of *Aronia melanocarpa*	200–400 mg/kg *Aronia melanocarpa* extract 200–400 mg/kg microencapsulated *Aronia melanocarpa*	6 weeks	*Aronia melanocarpa* was effective in preventing and slowing down atherosclerosis by increasing the activity of the PON1 enzyme, which plays a role in preventing lipid oxidation, the first step of atherosclerosis	[103]
Male Wistar aged rats (*n* = 24)	*Aronia melanocarpa* juice	10 mL/kg	3 month	↓Aging processes and oxidative stress in the testis ↑The functional activity of Leydig cells	[104]
Healthy male Wistar rats (*n* = 24)	*Aronia melanocarpa* juice	10 mL/kg	105 days	↑Improved myocardial nourishment and neovascularization ↑Improved age-related microvascular myocardial remodeling	[105]
Gentamicin‑induced Nephropathy Wistar rats (*n* = 30)	*Aronia melanocarpa* extract	3.38 mg/kg	30 days	↑ Protective effects on renal serum markers ↑ Neutrophil count	[106]
Ovariectomized female Wistar rats (*n* = 56)	*Aronia melanocarpa* fruit juice	5–10 mL/kg	3 month	Did not affect lipid accumulation and cholesterol levels ↑ Bone mineral density	[107]
Male Wistar aged rat hearts (*n* = 24)	*Aronia melanocarpa* fruit juice	440 mL	105 days	↓ Amount of collagen fibers in the coronary tunica media ↑ The intensity of the ACE2 enzyme	[108]
Pigs (*n* = 27)	*Aronia melanocarpa* pomace	NR	7 weeks	Intestinal barrier function upregulated the jejunal gene expression of ZO-1, Occludin, and Claudin-1.	[109]
High-fat diet and streptozotocin-induced T2DM male Wistar rats (*n* = 24)	*Aronia melanocarpa* extract	100–400 mg/kg	8 weeks	↓ Blood glucose, serum insulin levels, and the HOMA score ↑ Glucose metabolism enzyme activity ↓ Lipid accumulation, oxidative stress, and inflammation	[110]
Male Wistar rats with hyperuricemia (*n* = 60)	*Aronia melanocarpa* extract	0.5–2.0 g/kg	8 days	↑ Levels of glutathione peroxidase, superoxide dismutase, and catalase enzymes ↓ Serum levels of uric acid, blood urea nitrogen, and creatinine	[111]
Male Wistar albino rats with MetS (*n* = 60)	*Aronia melanocarpa* extract	0.45 mL/kg	4 weeks	↓ Blood pressure, oxidative stress, and liver steatosis ↑ Glucose tolerance	[112]

*T2DM* type 2 diabetes mellitus, *HOMA* homeostatic model assessment for insulin resistance, the angiotensin-converting enzyme 2 (*ACE2*), *MetS* Metabolic syndrom, *ZO-1,* Zonula Occludens-1, *NR* Not reported, *PON1* Paraoxonase 1

### Future Research Potential of *Aronia Melanocarpa*

In order to fully utilize the potential of *Aronia melanocarpa* in the food and pharmaceutical industries, it is of great importance that future research focuses on the mechanisms of action, safety, and efficacy of its active ingredients [4]. Strategies such as microencapsulation and nanotechnology are emerging as key areas of future studies to protect anthocyanins and other polyphenols, especially during processing and storage. Technological advances, especially in the fields of biotechnology and nanotechnology, will contribute to increasing the extraction efficiency of polyphenols while preserving their biological activity and improving the sensory properties of *Aronia melanocarpa-based* products [113, 114]. The development of formulations addressing health problems such as cardiovascular diseases, preventing diabetes, and supporting the immune system will further increase the market value of *Aronia melanocarpa*. With the increasing interest in natural and sustainable foods on a global scale, continuous optimization of extraction and processing technologies, utilization of not only the fruit but also other parts of the plant, and efficient use of resources, it is thought that the *Aronia melanocarpa* industry will increase its competitiveness and enter an economically and environmentally sustainable growth path.

## Conclusion

*Aronia melanocarpa* is a fruit rich in natural antioxidants, particularly anthocyanins, which contribute to its high antioxidant capacity. Studies indicate that consumption of *Aronia melanocarpa* may provide various health benefits, including modulation of oxidative stress, anti-inflammatory effects, and potential protective roles in metabolic and cardiovascular health. Additionally, *Aronia melanocarpa* extracts and formulations have shown promise in improving gut microbiota composition and supporting immune function. However, despite results from both in vitro and in vivo studies, clinical evidence in humans remains limited, and the optimal doses, forms, and duration of supplementation are yet to be clearly established. Incorporating *Aronia melanocarpa* into functional foods and supplements may support overall health, but personalized recommendations require further research. Future research, including well-designed human trials, exploration of nanoformulations, and mechanistic studies, is needed to fully elucidate *Aronia melanocarpa*’s health-promoting potential and to guide its application in functional foods and nutraceuticals.

## Data Availability

No datasets were generated or analysed during the current study.
